# Lea’s Medcial Epitome Series

**Published:** 1903-12

**Authors:** 


Lea's Medcial Epitome Series. Microscopy and Bacteriology. A Manual for Students and Practitioners. By P. E. Archinard, A. M., M. D., Demonstrator of Microscopy and Bacteriology, Tulane University of Louisiana, Medical Department. Duodecimo, pp. 210. Illustrated with 74 engravings. Series edited by V. C. Pedersen, A. M., M. D., Instructor in Surgery at the New York Polyclinic Medical School and Hospital. Lea Brothers & Co., Philadelphia and New York. 1903.

This contribution to the epitome series aims to afford to the student a clear and concise statement of the essentials of bacteriology and microscopy. Naturally, in a work of this character originality is neither expected nor desired. The book, however, meets all the requirements of a compend. Questions for quizzing are placed at the end of each chapter. It is an excellent little handbook for the beginner in bacteriological studies. M. J. F.

